# COVID-19 knowledge and practices in Jigawa State, Nigeria: A cross-sectional survey conducted during the second wave

**DOI:** 10.1371/journal.pgph.0003386

**Published:** 2024-07-01

**Authors:** Julius Salako, Damola Bakare, Abiodun Sogbesan, Tim Colbourn, Funmilayo Shittu, Ayobami A. Bakare, Obioma Uchendu, Hamish Graham, Eric D. McCollum, Agnese Iuliano, Rochelle Ann Burgess, James Beard, Adegoke G. Falade, Carina King, INSPIRING Consortium

**Affiliations:** 1 Department of Paediatrics, University of Ibadan, Ibadan, Nigeria; 2 Institute for Global Health, University College London, London, United Kingdom; 3 Department of Global Public Health, Karolinska Institutet, Stockholm, Sweden; 4 Department of Community Medicine, University College Hospital, Ibadan, Nigeria; 5 Department of Community Medicine, University of Ibadan, Ibadan, Nigeria; 6 Murdoch Children’s Research Institute, University of Melbourne Royal Children’s Hospital, Melbourne, Australia; 7 Department of Pediatrics, School of Medicine, Johns Hopkins University, Baltimore, United States of America; 8 Independant Consultant, United Kingdom; 9 Department of Paediatrics, University College Hospital, Ibadan, Nigeria; Ashoka University, INDIA

## Abstract

Population knowledge of COVID-19 and adherence to prevention measures may not be equitably distributed, limiting the success of public health measures. We aimed to understand whether COVID-19 knowledge differed by socio-economic status in a rural low-income setting of Jigawa State, Nigeria. We conducted a secondary analysis of the baseline cross-sectional survey of the INSPIRING cluster randomised controlled trial in Kiyawa Local Government Area, Jigawa State, from January—June 2021. Compounds were selected using simple random sampling proportional to trial cluster size. Within each compound, a representative head of compound and all women aged 16–49 years were eligible to complete a survey, which asked about socioeconomics, knowledge of COVID-19 symptoms, prevention strategies and risks for poor outcomes. We converted these into binary outcomes of “good knowledge” for symptoms, prevention and risks. Associations between woman and head of compound characteristics and good knowledge were assessed using adjusted logistic regression. We surveyed 3800 compound heads and 9564 women. Overall, <1% of respondents had been tested for COVID-19, but access to facemasks (HoC 60.0%; women 86.3%) and willingness to be vaccinated (HoC 73.9%; women 73.4%) were high. COVID-19 knowledge was low, with 33.2% of heads of compounds and 26.0% of women having good symptom knowledge, 39.5% and 30.4% having good prevention knowledge, and 17.7% and 15.4% having good risk knowledge, respectively. Those with more education, from higher wealth quintiles and access to a radio had better knowledge. Access to a mobile phone was associated with good symptom knowledge, but worse prevention and risk knowledge. We found significant differences in COVID-19 knowledge associated with socio-economic factors in rural Jigawa state, and access to communication devices was not consistently associated with better knowledge. Public health messaging in Nigeria needs to be adapted and delivered in way that ensures accessibility to all.

## Background

The earliest cases of the SARS-CoV-2 virus were detected in China in December 2019, with rapid spread to all regions of the world [1]. The World Health Organization (WHO) declared the COVID-19 pandemic in March 2020 due to its continuous global spread [2]. As of January 2024, over 774 million individuals had been confirmed as infected with over 7 million deaths [3] and 5 billion people fully vaccinated [4]. The SARS-CoV-2 virus spreads rapidly, with an incubation period of 2–14 days, and typically presents with a range of symptoms including dry cough, fever, tiredness, and shortness of breath or difficulty in breathing [5]. The reported case fatality rate during the pandemic varied from 0.1% to 25% between different countries and over time, depending on demographics, underlying co-morbidities, testing and reporting practices [6]. The majority of COVID-19 fatalities occur in older age groups and individuals with underlying medical conditions [7]. High numbers of COVID-19 cases placed significant burdens on healthcare systems globally during the pandemic phase [5].

As an airborne respiratory pathogen, key non-pharmaceutical interventions used to control COVID-19 spread included quarantine of suspected cases, contact tracing and travel restrictions, and later guidance on face masks, social distancing, hand washing with soap and isolation [8]. The pandemic saw widespread global “lockdowns”, which resulted in the shutdown of social activities in order to slow the spread of the disease, with consequences for national and regional economies [9].

Knowledge is a key factor that influences individual health behaviours towards different diseases [10]. The use of unproven treatments and the dissemination of false information about COVID-19, put the uptake and adherence to protective public health measures in jeopardy [11]. Therefore, successful control of COVID-19 was likely to be impacted by the population’s knowledge, attitudes, and practices towards the disease. As such, Government information campaigns need to be effective in informing people of policies, reasons for them, and simultaneously tackle misinformation. It is also crucial that information campaigns are inclusive, and accessible across society to promote equitable uptake of protective measures [12]. Several studies have been published on knowledge, attitudes, and practices toward COVID-19 in Nigeria [13–17]. However, knowledge varies between socioeconomic groups and there are gaps in understanding. According to Habib et al (2021), and Audu et al (2022), only 30.5% and 40.4% of participants respectively from Northern Nigeria had good knowledge of COVID-19 [16,18]. In other studies where knowledge was reported to be high, compliance to prevention practices was low [17,19].

Nigeria announced its first case of SARS-CoV-2 on the 27th February 2020 [20,21]. Nigeria, being the 7^th^ most populated country in the world, officially reported <1% of the global documented cases as of August 1, 2022 and only 0.05% of global deaths [22]. Modelling approaches suggest these numbers were vastly underestimated with the Institute for Health Metrics and Evaluation projecting over 140,000 COVID-19 deaths in Nigeria by January 2023–45 times higher than official numbers [23]. According to the Nigeria Center for Disease Control (NCDC), Lagos State was the epicentre, accounting for 39% of all cases and 24% of confirmed fatalities in Nigeria. The Northern region of Nigeria had fewer reported cases, with Jigawa State reporting the 5^th^ fewest cases, documenting just 18 fatalities and 669 cases for a population of approximately 7 million [24]. However, testing capacity in Jigawa and the northern region was low, and uptake of testing relies on the recognition of COVID-19 symptoms and motivation to test [25].

In reaction to the COVID-19 pandemic, the NCDC collaborated with states government, NGO’s and the private sector to support in the set-up isolation and treatment facilities for managing COVID-19 cases. For example, private sector were engaged to increase testing capacity, and NGOs in training of healthcare workers on case management, and infection prevention and control (IPC). The NCDC also coordinated trainings of healthcare workers on surveillance, risk communication, and other topics related to epidemic preparedness and response across all states [26]. In addition, as part of the COVID-19 response the NCDC set up information campaigns which included prevention and risks, with audio and visual social behavioural change communication (SBCC) materials. These were available in English and several different native languages across the 36 states of the federation, and the Federal capital of Abuja [27,28].

We therefore aimed to understand the knowledge of COVID-19 amongst adults in Jigawa State and whether knowledge differed between socio-economic groups. This will provide evidence on whether NCDCs and other organisations information campaigns effectively reached the different socio-economic groups in the context of Northern Nigeria, and to help to inform future public health responses to epidemics and pandemics.

## Methodology

We conducted a secondary analysis of cross-sectional data from women aged 16–49 years and heads of compounds, residing in Kiyawa Local Government Area (LGA), Jigawa State, Nigeria. Data were collected from January 14, 2021, to June 30, 2021 during the second wave of the pandemic in Nigeria [29], as part of a baseline survey for the INSPIRING Jigawa cluster randomised controlled trial (trial registration: ISRCTN 39213655, registered on 11^th^ December 2019) [30]. The primary outcome for the trial was child mortality, and the sample size of 12,160 for the baseline survey was based on detecting a 30% reduction in child deaths between intervention and control arms. We did not conduct a post-hoc power calculations for secondary analyses.

### Setting

There are a total of 11 wards in Kiyawa LGA, spread across three administrative districts, with a total estimated population of 230,000. Over 99% of the residents in Kiyawa LGA practice Islam, and agriculture is the main industry. The state’s sociocultural environment is homogeneous, dominated by the Hausa-Fulani and has high levels of poverty and child mortality [31].

### Study population

We included both heads of compounds and women of child-bearing age. The study eligibility criteria for head of compounds included any individual, not restricted by gender, who was identified by other compound members as being the head of the compound. For women, eligibility included those who were full-time residents of Kiyawa LGA and between the ages of 16 and 49. We conducted interviews with all women in this age group, regardless of their marital or gravid status.

### Sampling and recruitment

Compounds were sampled using simple random sampling, proportional to size based on the total number of compounds in the community (i.e. larger communities had more compounds visited). To develop the sample frame, we conducted a community mapping exercise during the formative phase of the INSPIRING project [30]. All compound numbers to be visited for each community were randomly sampled using Stata SE17 and pre-installed into the custom CommCare software used for data collection. There was no replacement for ineligible pre-selected sampled compounds. Within each compound, the head of compound and then all eligible women were recruited.

### Data collection procedures

We recruited and trained 23 female clinical data collectors who were all nurses, 8 male community mappers with a minimum of secondary school education, and 3 female and 2 male field supervisors who had a minimum of 10 years of working experience in both clinical and research settings. Data collectors worked in teams, with each team including a mapper who was responsible for identifying sampled compounds. All the data collectors (mapper, clinical data collectors, and supervisors) had 9 days of training which included both physical and remote sessions.

The clinical data collectors were responsible for conducting interviews with heads of compounds and women. The head of compound questionnaire included: compound structure, socio-demographics, COVID-19 knowledge and self-reported economic impacts of the COVID-19 pandemic (including loss of household income and loss of employment). All eligible women were administered a separate woman questionnaire which included: birth history, socio-demographics, care-seeking, pneumonia and COVID-19 knowledge, and community cohesion. In this paper we used the COVID-19 and socio-demographic sections of the questionnaires for both heads of compounds and women, and economic impacts for heads of compounds. The COVID-19 questions were developed by the study team and is not a validated tool, based on the official information being provided by the NCDC and WHO at the time of the survey–Table 1.

**Table 1:** Definitions of knowledge outcome variables.

COVID-19 knowledge variable	Criteria for” good” knowledge	Source of information
COVID-19 disease symptom	Can name cough, fever and difficulty breathing as symptoms of COVID-19	Nigeria Centre for Disease Control [32]
COVID-19 prevention strategies	Can mention at least 3 of the following 6 prevention strategies: wearing face mask, handwashing with soap, social distancing, avoiding crowded areas, disinfecting with alcohol, avoiding indoor areas.	World Health Organization 2020 [33]
COVID-19 risk of becoming very sick or dying	Can mention at least 2 of the following 8 characteristics of people who are at risk of becoming very sick or dying from COVID-19: older people, male gender, pregnant women, smokers, hypertension, diabetes, obesity and heart diseases.	Lieberman 2020, US Center for Disease Control 2023 [34,35]

We piloted the data collection tools between the 7^th^ to 12^th^ of January 2021 at Kwaimawa community, and this was done to ensure suitability, appropriateness, and adequacy of the contents among respondents. Kwaimawa community is located 4km away from Kiyawa LGA and residents are similar in socio-demographic characteristics with the people of Kiyawa LGA. All data was collected on Android tablets using CommCare (Dimagi, MA, USA).

### Analysis

Compound and woman characteristics were described using proportions and means, and COVID-19 knowledge data were converted into binary variables of “good knowledge” and “poor knowledge”–**Table 1**. The association between woman and head of compound characteristics and knowledge were tested using chi-squared tests and logistic regression, while adjusting for ownership of radio and mobile phone (p = 0.05). Data on COVID-19 testing, vaccination willingness and economic impacts were described using proportions. Analysis was conducted using Stata SE13 (StataCorp, TX, USA).

### Ethical approval

We obtained ethical approval from Jigawa State Government (ref: JPHCDA/ADM/GEN/073/V.I) and the University College London Research Ethics Committee (ref: 3433/004). Approval was also obtained from the Kiyawa Local Government Authority, District, Ward and Village heads of all communities before data collection in any community. The Head of Compound was asked for consent for the overall compound to be recruited into the study, and then individual informed verbal consent from all participants was sought before conducting interviews. A study data collector verbally explained the study using a standard consent form script. Participants were explicitly informed that participation is voluntary, and that the data collected would be used for research purposes only.

## Results

### Participant description

**Fig 1:**
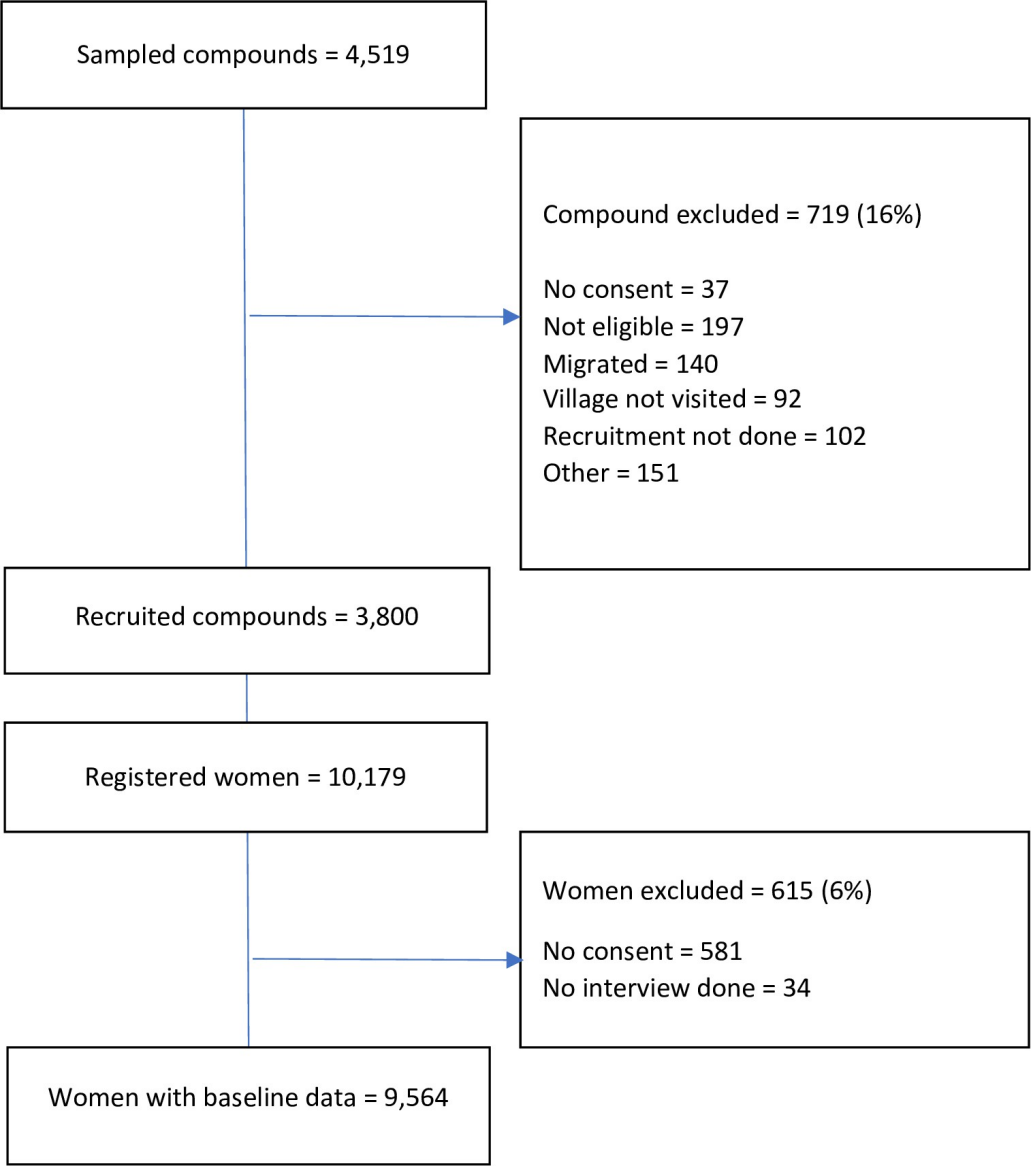
Participant inclusion diagram.

We recruited 9564 women from 3800 compounds (Fig 1). Table 2 shows a summary of eligible compound and woman characteristics. Of the 3800 compound heads, more than half (61.8%) had an informal or religious education and only 18.4% had formal education. Similar to what was observed amongst compound heads, 53.4% of women had informal or religious education and only 9.0% had a formal education. The majority (69.4%) of compound heads either own small businesses or practice subsistence farming as their major occupation while the majority of women (54.8%) practice skilled manual labour. Nearly all (96.3%) the women were married and 77.3% had a child under-5 years. The average number of eligible woman per compound was 3, with a mean age of 28.7 years, compared 57.1 years for compound heads.

**Table 2:** Socio-economic and demographic status of women and compound head.

Head of compound characteristics (N = 3800)		N	%
Age *(18 missing values)*	<40	586	15.5
	40–49	932	24.6
	50–59	935	24.7
	60–69	727	19.2
	70 and above	602	16.0
Gender	Male	3717	97.8
	Female	83	2.2
Education *(5 missing values)*	No formal education	752	19.8
	Informal/religious education	2345	61.8
	Formal education	698	18.4
Occupation *(13 missing values)*	Subsistence farming/ small business owner	2630	69.4
	Commercial farming/large business owner	410	10.9
	Unskilled manual labour	120	3.2
	Skilled manual labour	358	9.4
	Professional/ TBA	172	4.5
	Student/unemployed/retired	97	2.6
Wealth quintile	Lowest	765	20.1
	Low	783	20.6
	Middle	763	20.1
	High	733	19.3
	Highest	756	19.9
Women characteristics (N = 9564)		N	%
Age *(4 missing values)*	16–19	1447	15.1
	20–29	3846	40.2
	30–39	2663	27.9
	40–49	1605	16.8
Education *(11 missing values)*	No formal education	3593	37.6
	Informal/ Religious	5108	53.4
	Any formal education	852	9.0
Occupation	Business woman/Farming	2854	29.8
	Unskilled manual labour	425	4.4
	Skilled manual labour	5240	54.8
	Professional/TBA	21	0.3
	House work / not working Student	1024	10.7
Women with a child under-5 years	No under 5 child	2170	22.7
	Have an under 5	7394	77.3
Marital Status	Married	9214	96.3
	Never married	154	1.6
	Divorced/ Separated	142	1.5
	Widowed	54	0.6

TBA -Traditional birth attendant.

### Knowledge of COVID-19 among women and head of compounds

**Fig 2:**
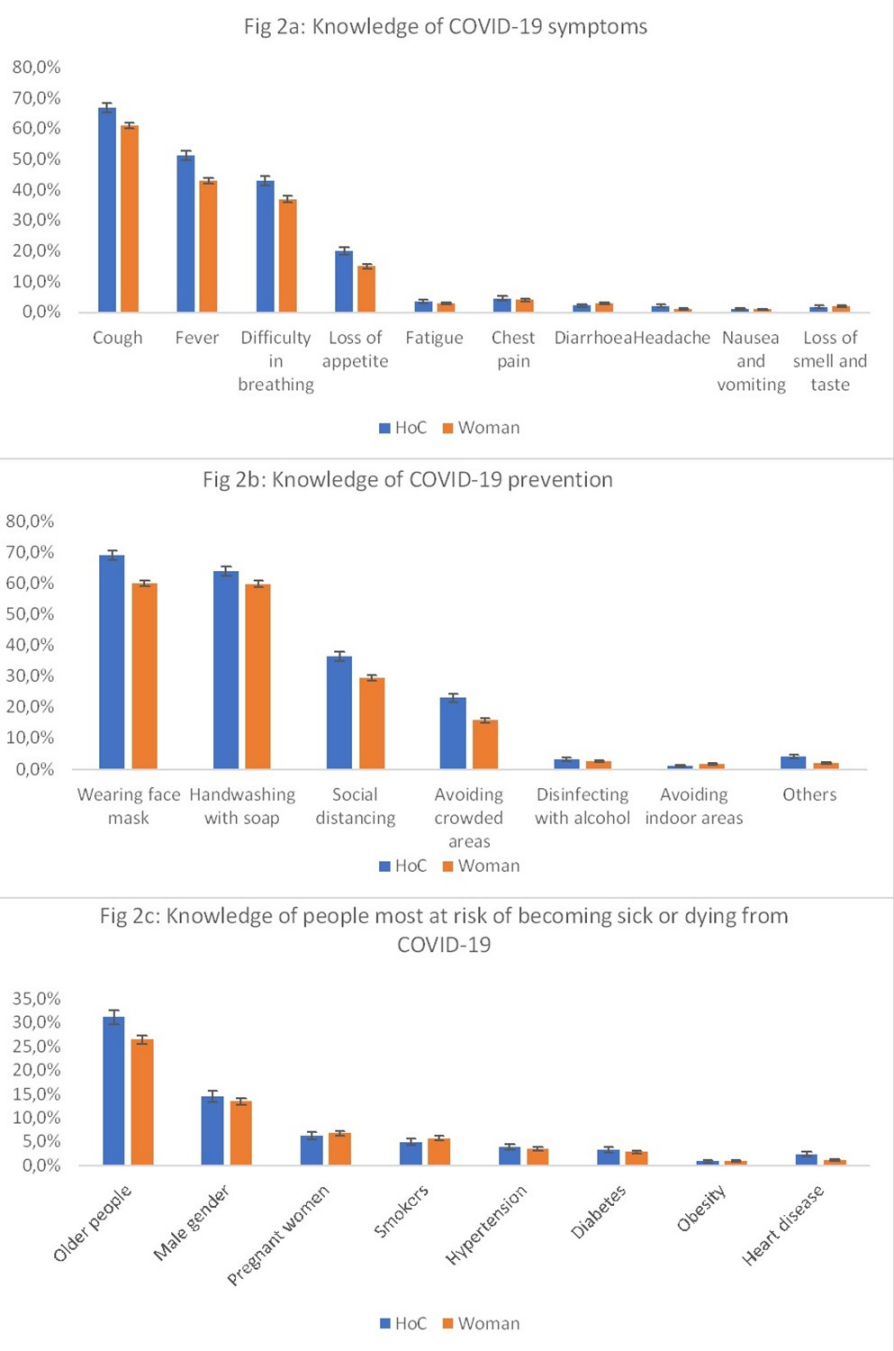
Difference in knowledge of COVID-19 between women and head of compound.

Knowledge of COVID-19 symptoms, prevention and risks were similar between women and heads of compounds–**Fig 2**. Cough (Head of Compound, HoC: 66.8% CI: 65.3–68.3, Women, W: 61.2% CI:60.2–62.2), fever (HoC: 51.2% CI: 49.6–52.8, W: 42.9% CI: 41.9–43.9), and difficulty in breathing (HoC: 42.9% CI: 41.4–44.6, W: 37.3% CI: 36.3–38.3) were the most frequently mentioned symptoms (Fig 2A). For ways to prevent COVID-19 infections, the use of face masks was the most frequently mentioned (HoC: 69.1% CI: 67.6–70.6, W: 60.1% CI: 59.1–61.1), followed by hand washing (HoC: 64.1% CI: 62.6–65.6, W: 59.9% CI: 58.9–60.9), social distancing (HoC: 36.4% CI: 34.9–37.9, W: 29.4% CI: 28.5–30.3)—Fig 2B. Knowledge about categories of people most at risk of becoming very sick or dying from COVID-19, was overall lower. Nearly a third of head of compounds (31.2% CI 29.7–32.7) and 26.5% (CI: 25.6–27.4) of women mentioned older people, followed by male gender (HoC: 14.6% CI: 13.5–15.7, W: 13.5% CI: 12.8–14.2); the least mentioned were heart disease and obesity with 1.0% response rate (Fig 2C).

We found 33.2% (1262/3800) of compound heads had good knowledge of COVID-19 symptoms, compared to 26.0% (2487/9564) of women. Similarly, 39.5% (1500/3800) of compound heads had a good knowledge of COVID-19 prevention compared to 30.4% (2908/9564) of women. The knowledge of people most at risk of becoming very sick or dying from COVID-19 was lower in both groups where only 17.7% of compounds heads and 15.4% of women able to mention at least two high risk groups (**Table 3**). COVID-19 symptom, prevention and risk factor knowledge differed in heads of compounds and women by education, occupation and wealth quintile groups, with a positive trend for those with higher level of education and wealth (p-value <0.001)–**Table 3**.

**Table 3:** Summary of COVID-19 knowledge according to head of compounds and woman characteristics.

		Head of Compound						Women					
		Correct symptom knowledge	P-value	Good prevention knowledge	P-value	Good risk knowledge	P-value	Correct symptom knowledge	p-value	Good prevention knowledge	p- value	Good risk knowledge	p-value
Overall		1262 (33.2)		1500 (39.5)		672 (17.7)		2487 (26.0)		2908 (30.4)		1475 (15.4)	
Age*	16–19							315 (21.2)	<0.001	377 (26.1)	<0.001	199 (13.8)	0.005
	20–29							981 (25.5)		1157 (30.1)		604 (15.7)	
	30–39	178 (30.4)	0.454	215 (36.7)	0.277	87 (14.9)	0.082	760 (28.5)		887 (33.3)		454 (17.1)	
	40–49	322 (34.6)		356 (38.2)		179 (19.2)		431 (26.9)		486 (30.3)		218 (13.6)	
	50–59	323 (34.6)		383 (41.0)		181 (19.4)							
	60–69	241 (33.2)		291 (40.0)		129 (17.8)							
	≥ 70	197 (32.9)		253 (42.0)		94 (15.6)							
Education	No formal education	195 (26.0)	<0.001	196 (26.1)	<0.001	138 (18.4)	<0.001	753 (21.0)	<0.001	789 (22.0)	<0.001	577 (16.1)	<0.001
	Informal/religious education	729 (31.1)		893 (38.1)		318 (13.6)		1351 (26.5)		1654 (32.4)		669 (13.1)	
	Formal education	338 (48.4)		409 (58.6)		216 (31.0)		382 (44.8)		465 (54.6)		229 (26.9)	
Occupation	Farming	611 (31.9)	<0.001	747 (39.0)	<0.001	268 (14.0)	<0.001	5 (20.0)	<0.001	8 (32.0)	<0.001	5 (20.0)	<0.001
	Unskilled manual labour	31 (25.8)		42 (35.0)		17 (14.2)		202 (47.5)		172 (40.5)		53 (12.5)	
	Skilled manual labour	113 (31.6)		123 (34.4)		80 (22.4)		1288 (24.6)		1615 (30.8)		837 (16.0)	
	Business owner/woman	376 (33.5)		439 (39.1)		240 (21.4)		816 (28.9)		885 (31.3)		484 (17.1)	
	Professional/TBA	101 (58.7)		111 (64.5)		51 (30.0)		11 (52.4)		14 (66.7)		6 (28.6)	
	Not working	30 (27.3)		38 (34.6)		16 (14.6)		165 (16.1)		214 (20.9)		90 (8.8)	
Wealth	Lowest	161 (21.1)	<0.001	250 (32.7)	<0.001	98 (12.8)	<0.001	182 (13.1)	<0.001	280 (20.2)		129 (9.3)	<0.001
	Low	192 (24.5)		244 (31.2)		80 (10.2)		314 (17.6)		402 (22.5)		184 (10.3)	
	Middle	207 (27.1)		260 (34.1)		113 (14.8)		359 (18.7)		451 (23.5)		215 (11.2)	
	High	314 (42.8)		325 (44.3)		179 (24.4)		609 (30.2)		686 (34.0)		374 (18.5)	
	Highest	388 (51.3)		421 (55.7)		202 (26.7)		1023 (41.7)		1089 (44.4)		573 (23.4)	
Access to radio	Yes	543 (47.2)	<0.001	600 (52.1)	<0.001	305 (26.5)	<0.001	1331 (36.0)	<0.001	1441 (39.0)	<0.001	776 (21.0)	<0.001
	No	719 (27.1)		900 (34.0)		367 (13.8)		1156 (19.7)		1467 (25.0)		699 (11.9)	
Access to mobile	Yes	1124 (34.6)	<0.001	1241 (38.2)	<0.001	543 (16.7)	<0.001	2307 (26.7)	<0.001	2508 (29.0)	<0.001	1262 (14.6)	<0.001
	No	138 (25.1)		259 (47.1)		129 (23.5)		180 (19.3)		400 (43.0)		213 (22.9)	

* There were no heads of compound aged under 30 and no women aged over 49 in our sample.

Overall, much fewer respondents had good knowledge across multiple dimensions of symptoms, prevention and risk, with only 9.2% for women having good symptom and risk knowledge–**S1 Table**. Only 10.9% of compound heads and 7.8% of women scored good in all 3 dimensions of COVID-19 knowledge.

### Associations between COVID-19 knowledge and socio-demographic factors

Results from adjusted logistics regressions on head of compound and woman characteristics, and good knowledge are presented in **Table 4** (the unadjusted model is presented in S2 Table). We observed an association between woman’s age and their knowledge of COVID-19 symptoms and prevention, but not risks–while older heads of compounds had increased odds of good prevention and risk knowledge. Increased wealth quintile was consistently associated with improved knowledge, across all domains for women and heads of compounds. Similarly, we found an association between education and knowledge, with those with any education (formal or informal) having better knowledge across all categories of knowledge, except risks, where informal or religious education was associated with poorer knowledge in both groups (HoC: AOR = 0.68, 95% CI: 0.54–0.85; W: AOR = 0.73, 95% CI: 0.64–0.82).

**Table 4:** Adjusted multivariable logistic regression analysis of COVID-19 knowledge and socio-demographic factors.

		Head of Compound				Women			
Variables		Adj. odds ratio	95% confidence interval		p-value	Adj. odds ratio	95% confidence interval		p-value
COVID-19 Symptoms Knowledge									
Age	16–19 years					ref			
	20–29 years					1.18	(1.01	1.37)	**0.031**
	30–39 years	ref				1.35	(1.15	1.59)	**<0.001**
	40–49 years	1.25	(0.98	1.58)	0.062	1.27	(1.06	1.52)	**0.009**
	50–59 years	1.19	(0.93	1.51)	0.150				
	60–69 years	1.05	(0.81	1.35)	0.697				
	70 years and above	1.08	(0.83	1.42)	0.537				
Occupation	Farming	ref				ref			
	Unskilled manual labour	0.60	(0.38	0.92)	**0.022**	3.48	(1.24	9.72)	**0.017**
	Skilled manual labour	0.79	(0.61	1.03)	0.084	1.25	(0.45	3.43)	0.659
	Business owner	0.83	(0.70	0.98)	**0.037**	1.42	(0.51	3.92)	0.490
	Professional/TBA	1.35	(0.93	1.94)	0.106	1.98	(0.51	7.72)	0.322
	Not working	0.64	(0.40	1.02)	0.065	0.81	(0.29	2.27)	0.700
Education	No formal education	ref				ref			
	Informal/religious education	1.26	(1.04	1.53)	**0.015**	1.24	(1.12	1.39)	**<0.001**
	Formal education	1.89	(1.48	2.42)	**<0.001**	2.34	(1.98	2.77)	**<0.001**
Wealth quintile	Lowest socioeconomic status	ref				ref			
	Low/Middle socioeconomic status	1.18	(0.90	1.53)	0.212	1.36	(1.10	1.69)	**0.004**
	Middle socioeconomic status	1.17	(0.90	1.52)	0.237	1.25	(1.01	1.55)	**0.033**
	Middle/High socioeconomic status	2.24	(1.72	2.92)	**<0.001**	2.25	(1.83	2.77)	**<0.001**
	Highest socioeconomic status	2.66	(2.00	3.54)	**<0.001**	3.18	(2.57	3.92)	**<0.001**
Mobile phone access		1.05	(0.83	1.34)	0.686	0.89	(0.74	1.08)	**0.252**
Radio access		1.64	(1.38	1.94)	**<0.001**	1.51	(1.35	1.69)	**<0.001**
**COVID-19 Prevention Knowledge**									
	16–19 years					ref			
	20–29 years					1.21	(1.04	1.40)	**0.010**
	30–39 years	ref				1.39	(1.19	1.62)	**<0.001**
	40–49 years	1.15	(0.92	1.45)	0.211	1.25	(1.05	1.48)	**0.011**
	50–59 years	1.25	(0.99	1.57)	0.054				
	60–69 years	1.14	(0.89	1.46)	0.280				
	70 years and above	1.41	(1.09	1.82)	**0.008**				
Occupation	Farming	ref				ref			
	Unskilled manual labour	0.75	(0.50	1.12)	0.165	1.44	(0.58	3.57)	0.426
	Skilled manual labour	0.65	(0.50	0.83)	**0.001**	0.96	(0.40	2.34)	0.945
	Business owner	0.79	(0.67	0.94)	**0.008**	0.94	(0.38	2.29)	0.902
	Professional/TBA	1.12	(0.77	1.63)	0.535	2.27	(0.60	8.51)	0.223
	Not working	0.65	(0.42	1.01)	0.058	0.65	(0.26	1.60)	0.352
Education	No formal education	ref				ref			
	Informal/religious education	1.72	(1.42	2.08)	**<0.001**	1.61	(1.45	1.78)	**<0.001**
	Formal education	3.32	(2.60	4.25)	**<0.001**	3.40	(2.88	4.01)	**<0.001**
Wealth quintile	Lowest socioeconomic status	ref				ref			
	Low/Middle socioeconomic status	1.29	(1.01	1.65)	**0.040**	1.85	(1.52	2.26)	**<0.001**
	Middle socioeconomic status	1.18	(0.92	1.51)	0.177	1.62	(1.33	1.97)	**<0.001**
	Middle/High socioeconomic status	1.86	(1.44	2.40)	**<0.001**	2.77	(2.27	3.38)	**<0.001**
	Highest socioeconomic status	2.45	(1.86	3.23)		3.77	(3.08	4.62)	**<0.001**
Mobile phone access		0.44	(0.36	0.56)	**<0.001**	0.27	(0.23	0.32)	**<0.001**
Radio access		1.58	(1.34	1.87)	**<0.001**	1.41	(1.26	1.57)	**<0.001**
**COVID-19 Risk Knowledge**									
Age	16–19 years					ref			
	20–29 years					1.11	(0.93	1.33)	0.239
	30–39 years	ref				1.18	(0.97	1.43)	0.083
	40–49 years	1.56	(1.16	2.10)	**0.003**	0.91	(0.73	1.13)	0.416
	50–59 years	1.54	(1.14	2.07)	**0.004**				
	60–69 years	1.44	(1.05	1.99)	**0.024**				
	70 years and above	1.34	(0.95	1.89)	0.090				
Occupation	Farming	ref				ref			
	Unskilled manual labour	0.91	(0.51	1.53)	0.742	0.52	(0.18	1.50)	0.227
	Skilled manual labour	1.52	(1.14	2.05)	**0.005**	0.64	(0.23	1.77)	0.392
	Business owner	1.37	(1.09	1.64)	**0.003**	0.63	(0.22	1.78)	0.393
	Professional/TBA	1.20	(0.83	1.86)	0.358	0.81	(0.19	3.38)	0.783
	Not working	0.89	(0.51	1.60)	0.699	0.34	(0.12	0.97)	**0.045**
Education	No formal education	ref				ref			
	Informal/religious education	0.68	(0.54	0.85)	**0.001**	0.73	(0.64	0.82)	**<0.001**
	Formal education	1.50	(1.14	1.98)	**0.003**	1.42	(1.18	1.72)	**<0.001**
Wealth quintile	Lowest socioeconomic status	ref				ref			
	Low/Middle socioeconomic status	1.12	(0.79	1.61)	0.506	1.85	(1.42	2.42)	**<0.001**
	Middle socioeconomic status	1.26	(0.90	1.76)	0.167	1.59	(1.22	2.05)	**<0.001**
	Middle/High socioeconomic status	2.41	(1.72	3.37)	**<0.001**	2.94	(2.27	3.80)	**<0.001**
	Highest socioeconomic status	2.21	(1.54	3.18)	**<0.001**	3.74	(2.88	4.87)	**<0.001**
Mobile phone access		0.40	(0.31	0.53)	**<0.001**	0.30	(0.24	0.37)	**<0.001**
Radio access		1.63	(1.33	2.00)	**<0.001**	1.45	(1.27	1.66)	**<0.001**

TBA: traditional birth attendant.

The relationship between occupation and knowledge was less consistent between knowledge domains and between heads of compounds and women. For example, women who are unskilled manual labour had good knowledge of symptom (W: AOR = 3.84, 95% CI: 1.24–9.72), whereas HoC in that job category were associated with poorer COVID-19 symptom knowledge (HoC: AOR = 0.60, 95% CI: 0.38–0.92). While access to a radio was consistently associated with improved knowledge, mobile phone access was associated with worse risk and prevention knowledge in both participant groups.

### Practices related to COVID-19

Less than 1% (39/9564) of women and 1.4% (54/3800) of the compound heads reporting having had a COVID-19 test. The majority of women (98.9%) were not sure if they have had a COVID-19 test, while most compound heads (98.1%) were certain they had not had a test (**Table 5**). All women who had tested said they tested negative, and only 1 of the 54 compound heads who had taken the test reported a positive test result. When asked about willingness to vaccinate, 73.9% of women and 73.4% of compound heads said they would be willing to receive a COVID-19 vaccine. More heads of compounds had access to a facemask than women (86.3% versus 60.0%) (**Table 5**).

**Table 5:** COVID-19 practices and perceptions among women and head of compounds.

		Woman (N = 9564)	Head of compound (N = 3800)
Had COVID-19 test*	Yes	39 (0.4)	54 (1.4)
	No	69 (0.7)	3729 (98.1)
	Don’t know	9456 (98.9)	17 (0.5)
Any other compound member had a COVID-19 test**	Yes		62 (1.6)
	No		3725 (98.0)
	Dont know		13 (0.4)
Access to any face mask	Yes	5743 (60.0)	3284 (86.3)
	No	3821 (40.0)	516 (13.7)
Willingness to get a COVID-19 vaccine	Yes No Dont know	7069 (73.9) 1260 (13.2) 1235 (12.9)	2790 (73.4) 406 (10.9) 604 (15.9)
When they wash hand with soap**	Wash hand before cooking	5782 (60.5)	
	Wash hand after toilet	7050 (73.7)	
	Wash hand after handling child faeces	5578 (58.3)	
	Wash hand after working in the garden	1923 (20.1)	
	Wash hand after looking after animal	1228 (12.8)	

*All tests were reported to be negative for women, and 1 positive test was reported among Head of Compounds.

**Questions only asked to one group of respondents.

### Economic impact of COVID-19

**Fig 3:**
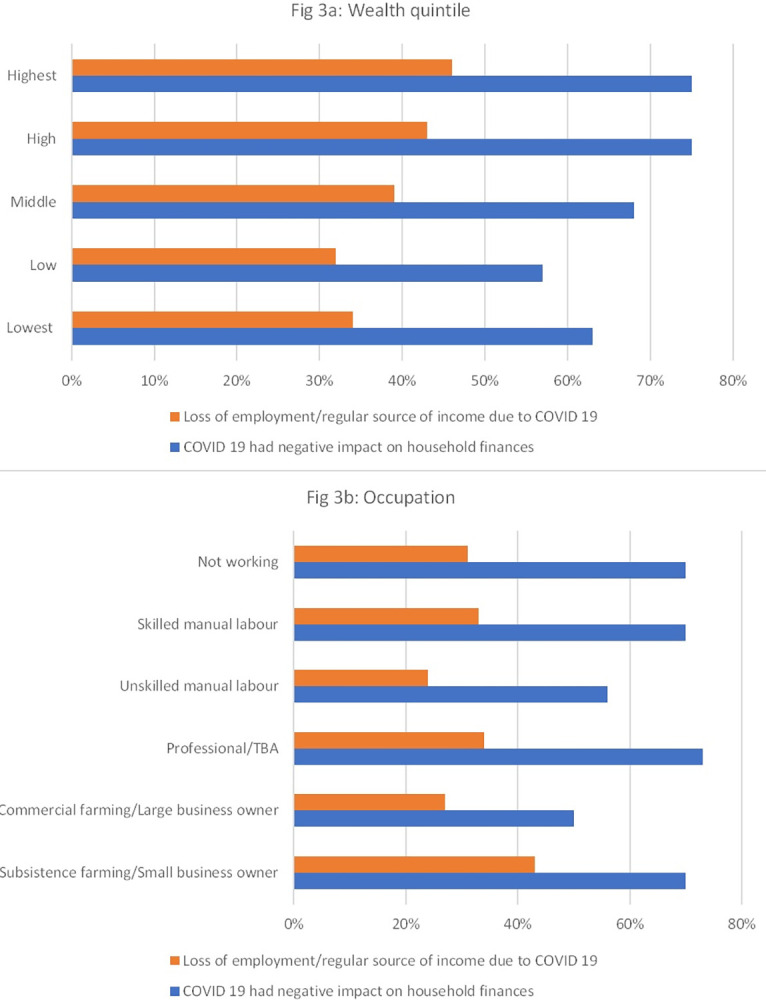
Self-reported impact of COVID-19 pandemic and response on compound income and loss of employment.

When asked if the COVID-19 pandemic had any negative effect on their livelihood, 67.5% (2564/3800) of head of compounds reported experiencing a negative impact on their household finances, and this proportion was higher amongst the compounds in the higher wealth quintiles—**Fig 3A**. In a similar fashion, the two highest wealth categories (high 43% and highest 46%) also reported the highest loss of employment/ loss of income due to COVID 19 (Fig 3B). While respondents from all occupation groups self-reported negative impacts of COVID-19, this was highest amongst professional groups (73%), small business owners, and skilled manual labours and not working (70% each).

## Discussion

We assessed COVID-19 knowledge and practices amongst heads of compounds and women in Kiyawa LGA, Jigawa State, Nigeria, and whether this was associated with socio-demographic factors. We found several factors were linked to knowledge, with approximately 1/3 participants having good knowledge of COVID-19 symptoms and prevention respectively, while knowledge of risks for poor outcomes was lower. We also found that a high proportion of compounds experienced a negative economic effect of the pandemic, and that willingness to vaccinate was high, despite confirmed cases in this setting being incredibly rare.

This survey found that the general level of knowledge on COVID-19 was low amongst the population, with similar findings amongst heads of compounds with the publication from Habib et al (2021) where only 30.5% of respondents in Northern Nigeria had good knowledge of COVID-19 [16]. We observed that people with formal education had a better general knowledge of COVID-19 compared to those who had Islamic education or no education at all. This reflects similar findings to research from Western Nigeria, where knowledge increased as education increased [36]. This association may not come as a surprise as education is a key determinant of health literacy and may also influence how well someone responds to survey questions. Formal education can also support individuals develop critical thinking skills and therefore be more critical in assessing reliability of information. Given the low rates of formal education in the population, it is important that communication approaches are tailored to the context, and presented in an accessible way [37].

We found that the role of someone’s occupation acted differently between the two respondent groups, when adjusting for wealth and education. Not working was consistently associated—although not always significantly, with worse knowledge, while being a business owner or doing manual labour acted in different ways. Anecdotally, it is plausible that those not working, largely retired people and housewives, had lower exposure to health messaging because they spend most of their time at home. Likewise, women who are skilled labourers such as tailors and cap weavers often ply their trade from home, and as such rely on information that reaches them at their homes. Women who practice unskilled manual labour on the other hand (such as grocery clerks, cleaners, food hawking, nannies, door to door house help) more commonly travel outside their homes for work. This group may therefore be exposed to more information sources, whereas unskilled manual labour for men include farm labourers, construction site workers, night guards, which may not expose them to information exchange and social interaction as much. Being a business owner had inconsistent patterns of association with knowledge, and it is unclear why knowledge for some domains would be higher or lower between heads of compounds and women. A study in South-East Nigeria found that traders, business owners, and people who are self-employed had poor knowledge of COVID-19 prevention practices [38], while other studies reported no associations [39,40]. The role of occupation is likely to be highly context specific, and reflective of broader cultural communication norms. While access to a radio was consistently associated with increased knowledge, suggesting this form of mass media was successful in reaching communities, we found that access to a mobile phone was associated with lower levels of prevention and risk knowledge. It may be that access to a mobile phone exposed individuals to social media, where sharing of COVID-19 misinformation was commonplace [41]. There is a clear need for reliable public health information that reaches all groups of people.

We found over 70% of participants were willing to receive COVID-19 vaccine if made available to them, very similar to the 72% reported by Oche et al. in Sokoto State [42]. Given historical boycotts of immunization programs in Northern Nigeria [43], and widespread rumours and misinformation about COVID-19 vaccines, we anticipated lower willingness to receive the COVID-19 vaccine [44]. Another study conducted between August and September 2021, just after our survey, reported that acceptance of the COVID-19 vaccine in Jigawa state was low, with 75% not believing in the safety of the vaccines, but that >60% would be willing to take the vaccine if it doesn’t harm those who have received it [45]. As of August 2023, just 35.6% of Nigerians had been fully immunized, far behind the WHO’s 70% vaccination coverage target by June 2022 [4]. While there have been challenges in COVID-19 vaccine supply, it also suggests that willingness has not translated into uptake. Mistrust in government and health care workers, misinformation and fear of side effects have all been identified as factors contributing to vaccine hesitancy in Nigeria [46,47]. Therefore, further research is needed to understand the relative contribution of contextual barriers to higher coverage.

COVID-19 was widely reported to have negatively impacted finances in Nigeria. In Edo State Nigeria, 36% of people reported a high negative effect of COVID-19 pandemic on their livelihood [48], and in Adamawa, Kogi and Oyo state, 83.3% of respondents reported a negative change in customer patronage and 86.6% reported that their business became indebted due to perishable goods in the lockdown period [49]. In a report by the Jigawa State Internal Revenue Board, the chairman disclosed that the state lost over 20% of the internally generated revenue from 2019 due to tax relief policies instituted during COVID-19 [50]. This ranges from loss of employment to loss of a regular source of income. In order to reduce the spread of this disease, the Federal and State governments declared lockdowns, with markets, schools and religious gatherings prohibited [51,52]. People who earn their sources of income in any of these settings would likely be negatively affected. We observed that those in higher wealth quintiles reported these negative impacts more but may be more reflective of those in lower wealth quintiles already experiencing extreme poverty without a stable income. Other studies that supports our findings include but not limited to Afolabi 2021, and UNDP 2021 [53,54].

We found a significant number of women reported not having access to soap or washing their hands regularly. Habib 2021, reported that overall COVID-19 knowledge on prevention such as use of facemasks, social distancing and washing of hands with soap and water was good, but did not translate to actual practice [16]. Linda et al. 2022 reported that even though nurses had good knowledge of COVID-19 preventive measures, level of compliance to practice of preventive measures was low [55].

We had several limitations in this study, firstly given the cross-sectional design, we cannot draw any conclusions on the direction of associations and many of the practices were self-reported and therefore subject to recall and social desirability biases. Secondly, when we asked if participants had had a COVID-19 test, most women responded that they were not sure, while heads of compounds were confident to respond. This may indicate that the questionnaire was poorly understood, despite piloting. However, given the low testing rates, this could also indicate that information on COVID-19 testing in this setting was poor. Finally, this represents knowledge and practices at a particular point in time; given recommendations and understanding of COVID-19 rapidly evolved, we were unable to explore how knowledge and practices adapted to new information.

## Conclusion

Despite widespread initiatives taken by the Nigerian government and NCDC, including mass media campaigns, knowledge of COVID-19 was low and not evenly spread amongst a general representative population of rural Jigawa. There is a need for public health stakeholders to reflect on diverse community experiences from COVID-19, where both the disease itself and the control measures had negative consequences to differing degrees. In our study, it was clear that negative economic impacts were widespread, while burden and knowledge of the disease was low–a potentially dangerous mix for ensuring population compliance with restrictions, and therefore pandemic containment. Mass awareness campaigns should be designed together with rural and under-served communities, particularly considering the role of religious and traditional leaders in supporting the dissemination of accurate and accessible information. Given the lack of access to common forms of mass media, such as radios, phones and televisions, active outreach and integrating with existing community structures, is needed to ensure reliable information is available to all.

## Supporting information

S1 Table Cross tabulation of knowledge categories. *Yellow = Hoc (N = 3800), Blue = women (N = 9564).
(DOCX)

S2 Table Unadjusted logistic regression analysis of COVID-19 knowledge. TBA = Traditional birth attendant.
(DOCX)

S1 Text Inclusivity in global research questionnaire. (PDF)
