# Supplementary material for: COVID-19 knowledge and practices in Jigawa State, Nigeria: A cross-sectional survey conducted during the second wave
Source: PLOS Glob Public Health. 2024 Jul 1;4(7):e0003386. doi: 10.1371/journal.pgph.0003386 (PMC11216585; doi:10.1371/journal.pgph.0003386)
Supplement: S1 Table — *Yellow = Hoc (N = 3800), Blue = women (N = 9564). (DOCX) [file pgph.0003386.s001.docx]

**S1 Table: Cross tabulation of knowledge categories**

|  | **Symptoms** | **Prevention** | **Risks** |
| --- | --- | --- | --- |
| **Symptoms** |  | 24.4% (<0.001) | 12.4% (<0.001) |
| **Prevention** | 18.4% (<0.001) |  | 14.6% (<0.001) |
| **Risks** | 9.2% (<0.001) | 11.4% (<0.001) |  |
|  | | | |
| **All domains** | 7.8% (<0.001) | 10.9% (<0.001) |  |

*Yellow = Hoc (N= 3800), Blue = women (N=9564)
