# Supplementary material for: COVID-19 knowledge and practices in Jigawa State, Nigeria: A cross-sectional survey conducted during the second wave
Source: PLOS Glob Public Health. 2024 Jul 1;4(7):e0003386. doi: 10.1371/journal.pgph.0003386 (PMC11216585; doi:10.1371/journal.pgph.0003386)
Supplement: S2 Table — TBA = Traditional birth attendant. (DOCX) [file pgph.0003386.s002.docx]

**S2 Table: Unadjusted logistic regression analysis of COVID-19 knowledge**

|  | | **Head of Compound** | | | | **Women** | | | |
| --- | --- | --- | --- | --- | --- | --- | --- | --- | --- |
| **Variables** | | **Odds ratio** | **95% confidence interval** | | **p-value** | **Odds ratio** | **95% confidence interval** | | **p-value** |
| **COVID-19 Symptoms Knowledge** | |  |  |  |  |  |  | |  |
| Age | 16-19 years |  |  |  |  | ref |  |  |  |
|  | 20-29 years |  |  |  |  | 1.17 | (1.01 | 1.37) | **0.036** |
|  | 30-39 years | ref |  |  |  | 1.37 | (1.17 | 1.61) | **<0.001** |
|  | 40-49 years | 1.33 | (1.05 | 1.68) | **0.016** | 1.31 | (1.10 | 1.57) | **0.003** |
|  | 50-59 years | 1.30 | (1.03 | 1.65) | **0.025** |  |  |  |  |
|  | 60-69 years | 1.16 | (0.90 | 1.49) | 0.231 |  |  |  |  |
|  | 70 years and above | 1.18 | (0.91 | 1.55) | 0.202 |  |  |  |  |
| Occupation | Farming | ref |  |  |  | ref |  |  |  |
|  | Unskilled manual labour | 0.57 | (0.37 | 0.89) | **0.014** | 3.72 | (1.33 | 10.43) | **0.012** |
|  | Skilled manual labour | 0.77 | (0.60 | 1.00) | 0.051 | 1.28 | (0.46 | 3.54) | 0.623 |
|  | Business woman | 0.81 | (0.68 | 0.96) | **0.015** | 1.48 | (0.53 | 4.09) | 0.445 |
|  | Professional_TBA | 1.36 | (0.94 | 1.96) | 0.094 | 2.13 | (0.54 | 8.32) | 0.275 |
|  | Not working | 0.65 | (0.41 | 1.03) | 0.070 | 0.86 | (0.30 | 2.39) | 0.774 |
| Education | No formal education | ref |  |  |  | ref |  |  |  |
|  | Informal/religious education | 1.26 | (1.04 | 1.52) | **0.016** | 1.23 | (1.10 | 1.37) | **<0.001** |
|  | Formal education | 1.91 | (1.49 | 2.44) | **<0.001** | 2.29 | (1.94 | 2.70) | **<0.001** |
| Wealth quintile | Lowest socioeconomic status | ref |  |  |  | ref |  |  |  |
|  | Low/Middle socioeconomic status | 1.22 | (1.49 | 2.44) | 0.103 | 1.32 | (1.08 | 1.62) | **0.006** |
|  | Middle socioeconomic status | 1.41 | (1.49 | 2.44) | **0.005** | 1.42 | (1.16 | 1.72) | **<0.001** |
|  | Middle/High socioeconomic status | 2.79 | (1.49 | 2.44) | **<0.001** | 2.65 | (2.20 | 3.20) | **<0.001** |
|  | Highest socioeconomic status | 3.56 | (1.49 | 2.44) | **<0.001** | 3.97 | (3.32 | 4.76) | **<0.001** |
| **COVID-19 Prevention Knowledge** | |  |  |  |  |  |  | |  |
| Age | 16-19 years |  |  |  |  | ref |  |  |  |
|  | 20-29 years |  |  |  |  | 1.22 | (1.05 | 1.40) | **0.006** |
|  | 30-39 years | ref |  |  |  | 1.44 | (1.24 | 1.68) | **<0.001** |
|  | 40-49 years | 1.17 | (0.94 | 1.47) | 0.153 | 1.32 | (1.11 | 1.56) | **0.001** |
|  | 50-59 years | 1.33 | (1.06 | 1.67) | **0.012** |  |  |  |  |
|  | 60-69 years | 1.23 | (0.97 | 1.57) | 0.080 |  |  |  |  |
|  | 70 years and above | 1.47 | (1.14 | 1.90) | **0.002** |  |  |  |  |
| Occupation | Farming | ref |  |  |  | ref |  |  |  |
|  | Unskilled manual labour | 0.73 | (0.49 | 1.09) | 0.133 | 1.44 | (0.59 | 3.50) | 0.414 |
|  | Skilled manual labour | 0.66 | (0.52 | 0.85) | **0.001** | 0.99 | (0.41 | 2.36) | 0.992 |
|  | Business woman | 0.79 | (0.67 | 0.93) | **0.006** | 0.97 | (0.40 | 2.31) | 0.953 |
|  | Professional_TBA | 1.16 | (0.80 | 1.68) | 0.416 | 2.20 | (0.60 | 8.09) | 0.232 |
|  | Not working | 0.67 | (0.43 | 1.03) | 0.071 | 0.66 | (0.27 | 1.58) | 0.353 |
| Education | No formal education | ref |  |  |  | ref |  |  |  |
|  | Informal/religious education | 1.71 | (1.42 | 2.06) | **<0.001** | 1.60 | (1.45 | 1.77) | **<0.001** |
|  | Formal education | 3.44 | (2.70 | 4.38) | **<0.001** | 3.45 | (2.93 | 4.07) | **<0.001** |
| Wealth quintile | Lowest socioeconomic status | ref |  |  |  | ref |  |  |  |
|  | Low/Middle socioeconomic status | 0.90 | (0.72 | 1.12) | 0.368 | 1.05 | (0.88 | 1.26) | 0.519 |
|  | Middle socioeconomic status | 1.04 | (0.83 | 1.29) | 0.714 | 1.13 | (0.96 | 1.35) | 0.135 |
|  | Middle/High socioeconomic status | 1.54 | (1.24 | 1.92) | **<0.001** | 1.84 | (1.56 | 2.17) | **<0.001** |
|  | Highest socioeconomic status | 2.09 | (1.66 | 2.63) | **<0.001** | 2.52 | (2.15 | 2.96) | **<0.001** |
| **COVID-19 Risk Knowledge** | |  |  |  |  |  |  | |  |
| Age | 16-19 years |  |  |  |  | ref |  |  |  |
|  | 20-29 years |  |  |  |  | 1.12 | (0.94 | 1.34) | 0.196 |
|  | 30-39 years | ref |  |  |  | 1.22 | (1.01 | 1.48) | **0.031** |
|  | 40-49 years | 1.60 | (1.20 | 2.15) | **0.001** | 0.97 | (0.78 | 1.20) | 0.793 |
|  | 50-59 years | 1.67 | (1.25 | 2.24) | **0.001** |  |  |  |  |
|  | 60-69 years | 1.59 | (1.16 | 2.18) | **0.004** |  |  |  |  |
|  | 70 years and above | 1.43 | (1.02 | 2.01) | **0.037** |  |  |  |  |
| Occupation | Farming | ref |  |  |  | ref |  |  |  |
|  | Unskilled manual labour | 0.89 | (0.51 | 1.53) | 0.686 | 0.53 | (0.18 | 1.51) | 0.240 |
|  | Skilled manual labour | 1.53 | (1.14 | 2.05) | **0.004** | 0.66 | (0.24 | 1.80) | 0.418 |
|  | Business woman | 1.34 | (1.09 | 1.64) | 0.005 | 0.65 | (0.24 | 1.80) | 0.419 |
|  | Professional_TBA | 1.24 | (0.83 | 1.86) | 0.282 | 0.79 | (0.19 | 3.22) | 0.746 |
|  | Not working | 0.90 | (0.51 | 1.60) | 0.738 | 0.35 | (0.12 | 0.98) | **0.046** |
| Education | No formal education | ref |  |  |  | ref |  |  |  |
|  | Informal/religious education | 0.69 | (0.55 | 0.86) | **0.001** | 0.74 | (0.65 | 0.84) | **<0.001** |
|  | Formal education | 1.61 | (1.23 | 2.11) | **0.001** | 1.48 | (1.23 | 1.78) | **<0.001** |
| Wealth quintile | Lowest socioeconomic status | ref |  |  |  | ref |  |  |  |
|  | Low/Middle socioeconomic status | 0.72 | (0.52 | 0.99) | 0.050 | 1.07 | (0.84 | 1.36) | 0.548 |
|  | Middle socioeconomic status | 1.06 | (0.79 | 1.43) | 0.682 | 1.16 | (0.92 | 1.46) | 0.207 |
|  | Middle/High socioeconomic status | 1.88 | (1.42 | 2.48) | **<0.001** | 2.04 | (1.64 | 2.53) | **<0.001** |
|  | Highest socioeconomic status | 1.76 | (1.32 | 2.36) | **<0.001** | 2.59 | (2.10 | 3.20) | **<0.001** |
